# Cardiac Indices Parameters on the Ultrasonic Cardiac Output Monitor as Potential Indicators to Predict the Ultrafiltration Endpoint Success in Acute Heart Failure Treatment

**DOI:** 10.31083/RCM27100

**Published:** 2025-05-22

**Authors:** Yiou Li, Jiajia Chen, Jianye Bian, Fangyuan Chen, Qianli Wan, Fang Yuan

**Affiliations:** ^1^Department of Critical Care Medicine, Tongren Hospital Shanghai Jiao Tong University School of Medicine, 200336 Shanghai, China

**Keywords:** ultrasonic cardiac output monitor, acute decompensated heart failure, brain natriuretic peptide, ultrafiltration, echocardiography

## Abstract

**Background:**

Ultrafiltration (UF) is an alternative approach to diuretic therapy for the treatment of acute heart failure (AHF), but its optimal endpoint is unclear. This study explores using non-invasive ultrasonic cardiac output monitor (USCOM) to determine UF endpoints based on hemodynamic changes.

**Methods:**

In this single-anonymized, randomized controlled trial, acute decompensated heart failure patients were randomly assigned to UF (U, n = 20) and USCOM+UF (UU, n = 20) groups at a ratio of 1:1. A mixed linear model was utilized to analyze repeated measurement data of hemodynamic indicators (primary endpoint) in the U and UU groups. A 30% or 50% decrease in B-type natriuretic peptide (BNP) concentrations relative to the baseline was established as the criteria for the UF endpoint success. Multivariate logistic regression was used to identify potential indicators within the USCOM that could have influenced the UF endpoint success. Receiver operating characteristic (ROC) curves were used to evaluate the value of the predictive model. Economic benefits, including treatment costs and hospitalization duration, were also assessed.

**Results:**

Change rates in mean arterial pressure, heart rate (HR), urine output, hematocrit, and BNP concentrations were similar between the U and UU groups over 7 days (all *p* > 0.05). On day 4, significant correlations were found between various USCOM parameters, including inotropy (INO), systemic vascular resistance index (SVRI), systemic vascular resistance, corrected flow time (FTc), velocity time integral, and the BNP of the UF parameters. Multivariate logistic regression revealed that INO and SVRI were correlated with a 30% reduction in BNP on day 4 compared to baseline, while FTc and HR were found to be independently associated with a 50% reduction in BNP on day 4 compared to baseline. The UF endpoint prediction formula for a 30% reduction in BNP was –2.462 + 0.028 × INO – 0.069 × SVRI, with sensitivities, specificities, and accuracies of 70%, 83%, and 75%, respectively. The UF endpoint prediction formula for a 50% reduction of BNP was –2.640 – 0.088 × FTc – 0.036 × HR, with sensitivities, specificities, and accuracies of 83%, 63.0%, and 72.5%, respectively. The addition of the USCOM significantly reduced treatment costs and hospitalization stay lengths.

**Conclusions:**

Observing the USCOM using probability formulas served to determine appropriate UF endpoints during AHF treatments. UF combined with the USCOM can reduce the costs of UF and hospitalization.

**Clinical Trial Registration:**

NCT06533124, https://clinicaltrials.gov/study/NCT06533124?term=NCT06533124&rank=1.

## 1. Introduction

Acute heart failure (AHF) is a clinical syndrome caused by acute onset or exacerbation of left ventricular dysfunction, leading to decreased myocardial contractility and an increased load on the heart. This syndrome results in an abrupt decrease in cardiac output (CO) and an increase in pulmonary circulation pressure and peripheral vascular resistance, leading to acute pulmonary congestion, pulmonary edema and clinical symptoms that may be accompanied by inadequate tissue and organ perfusion resulting in cardiogenic shock [1, 2]. The etiology of AHF is undoubtedly complex, but is believed to be related to hemodynamic disturbances [3, 4]. A recent report found that the in-hospital mortality rate of AHF was 3%, and the 3 and 5 year mortality rates were 30% and 60%, respectively [5], indicating poor prognosis and high mortality. Pharmacological interventions aimed at achieving rapid decongestion and improving organ perfusion include positive inotropic drugs that increase CO, raising blood pressure, alleviation of tissue hypoperfusion and maintenance of the functions of vital organs [6]. Moreover, the use of diuretics for reducing congestion has been a major treatment mode for AHF patients in clinical practice [7], but about 20% of patients did not exhibit improved symptoms after treatment with diuretic drugs, and furthermore diuretic resistance occurred in >30% of them [8, 9].

Ultrafiltration (UF) is an alternative approach to diuretic therapy for congestion management, according to the European Society of Cardiology Guidelines for the diagnosis and treatment of AHF and chronic heart failure [3]. It is advised for patients with obvious volume overload in order to alleviate congestive symptoms and fluid weight (Class IIb, Level of Evidence: B). It has also been found to improve long-term outcomes for patients with acute decompensated heart failure (ADHF) [10]. The use of UF was shown to have little effect on all-cause mortality over the longest follow-up periods studied, but UF reduced all-cause re-hospitalization rates to ≤30 days and at the longest available follow-up [11], which may be related to the fact that UF therapy could more significantly alleviate volume overload. However, UF may lead to a decline in renal function, mainly manifested as elevated concentrations of serum urea and creatinine, as well as an increased risk of renal failure, bleeding and other complications [12, 13]. Another study also reported that among patients with ADHF who had worsened renal function and persistent congestion, the occurrence of serious adverse events was higher in those receiving UF treatment compared with those treated with pharmacological therapy [14]. Moreover, the duration of UF has been shown to be related to the cost of hospitalization of patients, and if UF is continued after attainment, it can further increase the financial burden on patients. Therefore, it would be of great clinical interest to have a predictor of when a patient has reached the endpoint of UF via hemodynamic changes. Hence, the alternative strategy for monitoring UF attainment by one non-invasive device is an endeavor of practical clinical relevance.

The ultrasonic cardiac output monitor (USCOM) 1A system (USCOM Ltd., Sydney, Australia) is a non-invasive Doppler stroke volume (SV) technique derived from echocardiography, that has been validated for a CO of 0.12 L/min to 18.3 L/min [15]. Moreover, it has the advantages of high repeatability, continuous monitoring and cost-effectiveness, and is especially suitable for predicting hemodynamic changes [16]. Furthermore, there are indicators on USCOM that reflect the volume, such as corrected flow time (FTc) and the systemic vascular resistance index (SVRI) [17, 18]. Compared with traditional invasive monitoring methods, such as pulse indicator continuous CO and Swan-Ganz floating catheters, USCOM can also obtain accurate and reliable data, and has been verified for both adult and pediatric patients [19, 20]. USCOM is easy to operate and trainees reached the same level as trainers after 50 operations, with the learning curve for skill acquisition being significantly shorter [21, 22]. USCOM combined with UF is a valuable tool for cardiologists to diagnose and manage the burden of body fluids, where adjusting UF periods according to USCOM data may well reduce the costs of AHF treatments. However, it remains a challenge to integrate optimally USCOM metrics with UF parameters to determine the exact timing of effective and timely monitoring of the UF endpoint.

The detection of B-type natriuretic peptide (BNP) or N-terminal pro-BNP (NT-proBNP) is recommended for screening, diagnosis and differential diagnosis of AHF, as well as for the assessment of the severity and prognosis of AHF [23, 24]. The variations of BNP before discharge have been independently associated with an increased risk of cardiovascular events, re-hospitalization or death after discharge [25]. At present, the Chinese guideline recommends UF to be applied for 7 consecutive days and BNP concentrations should be reduced by at least 50% compared to baseline [26]. Studies have shown that 30% and 46% declines in BNP at discharge are favorable values for the prognosis of heart failure patients [10, 27]. Other authors proposed a BNP/NT-proBNP reduction ≥30% as the standard for effective treatment, and a decrease of BNP/NT-proBNp <30% during hospitalization for AHF as indicative of an increased risk of re-hospitalization and death [28]. Thus, thresholds of a 30% and 50% reduction in BNP concentrations relative to baseline during the UF process were set as criteria to identify one or more potential indicators on the USCOM monitor and to establish a predictive model in the present study.

It would be of great clinical interest to have a predictor of when a patient has reached the endpoint of UF via hemodynamic changes. Therefore, the present single-blind, randomized control trial was designed to evaluate the feasibility of USCOM for determining UF endpoints during AHF treatments and to establish whether it is useful in reducing the financial burden on ADHF patients requiring UF. The primary objective of the trial was to evaluate differences in hemodynamics of patients receiving UF alone (U group) or UF + USCOM (UU group). The secondary objective was to construct a prediction model of potential indicators on USCOM (i.e., inotropy (INO), FTc, SVRI) for achieving UF standards based on threshold criteria of a 30% or 50% decrease in BNP concentration relative to baseline.

## 2. Materials and Methods

### 2.1 Study Design

This study was a single-blind, randomized controlled trial where ADHF patients were randomly assigned to U (n = 20) and UU (n = 20) groups at a ratio of 1:1. UF alone and UF + USCOM treatments from Day 1 to Day 7 were monitored. Repeated measurement data of hemodynamic indicators (primary endpoint) in U and UU groups were collected. A 30% or 50% decrease in BNP concentrations relative to baseline was set as the criteria for achieving UF-endpoint. Detailed information are shown in **Supplementary Fig. 1**.

### 2.2 Patients

This trial was based on the American College of Cardiology, American Heart Association, and Heart Failure Society of America guidelines for the management of AHF [29] and involved 40 patients diagnosed with ADHF from January 2022 to July 2023. Patients were randomly assigned to U (n = 20) and UU (n = 20) groups.

The inclusion criteria were: (1) age ≥18 years; (2) male or non-pregnant female patients; and (3) clinical symptoms or signs of fluid overload, among which fluid overload was defined as having met at least two of the following criteria: (a) pitting edema ≥2+ of the lower extremities; (b) moist rales in the lungs; (c) jugular venous distention >10 cm; (d) pulmonary edema or pleural effusion on chest X-ray; (e) paroxysmal nocturnal dyspnea or ≥ two-pillow orthopnea; (f) congestive hepatomegaly or ascites; and (g) BNP >400 pg/mL.

The exclusion criteria were: (1) hematocrit (HCT) >45%; (2) systolic blood pressure ≤90 mmHg and poor peripheral circulation; (3) contraindications to heparin anticoagulation; (4) renal insufficiency with a serum creatine ≥3.0 mg/dL or planned renal replacement therapies; (5) acute coronary syndromes; (6) life-threatening organ dysfunction caused by a dysregulated host response to infection; (7) active myocarditis; (8) patients with heart failure attributed to restrictive or hypertrophic cardiomyopathy or uncorrected valvular stenotic disease; (9) infection; (10) malignancies; (11) systemic immune disease; (12) unwillingness to cooperate; and (13) withdrawal from the study or death. The study adhered to the principles of the Declaration of Helsinki and the protocols were approved by the Institutional Review Board of our hospital (2021-072-01). Informed consent was obtained from all enrolled patients. This study was registered with ClinicalTrials.gov (identifier: NCT06533124).

### 2.3 Randomization and Masking

This study employed a randomized and single-blind design, where the randomization approach was “static randomization” and no stratification factors were set. The allocation of study participants was processed through an interactive web response system (IWRS), with a non-stratified permutated block size of 4. Patients were randomly assigned in a 1:1 ratio, with one group receiving UF treatment alone and the other receiving UF treatment plus USCOM monitoring. The statistician responsible for randomization set the randomization parameters in the background of the IWRS in advance. The IWRS generated the random allocation table, and the codes of the treatment regimens were also input into the system background. The study coordinator was responsible for obtaining the random number and corresponding treatment regimen through the IWRS and communicating the assignment information to the relevant investigator, in which, treating clinicians and the enrolled patients were unaware of group assignments.

### 2.4 The Operator Steps of USCOM

The operation of USCOM only requires the placement of the probe in the patient’s pulmonary artery or aortic window for monitoring. In pulmonary artery window monitoring, the probe is positioned beside the right sternal border or upper abdomen to assess blood flow in the pulmonary artery, thereby monitoring the pulmonary circulation and right heart function. For aortic window monitoring, the probe is placed at the sternal notch or subclavian fossa (same as the pulmonary artery window), and measurements are taken from the aorta to assess the systemic circulation, primarily monitoring left ventricular output. USCOM is easy to operate and trainees reached the same level as trainers after about 50 operations, with the learning curve for skill acquisition being significantly shorter [21]. In the present trial, all patients in the UU group were monitored by the same skilled operator and three consecutive measurements were made with a deviation of no more than 10% each time, in order to ensure the consistency and reliability of the data.

### 2.5 Assessments

Patients were assessed at baseline and throughout the period of treatment. The documented variables were medical history, physical examination data, echocardiography, laboratory blood test monitoring (continuous), including the total UF volume, body weight, patient symptoms, body position, transcutaneous oxygen saturation (SpO_2_), degree of edema, leg circumference, abdominal circumference, input-output balance and other variables for 7 days. Adverse events were assessed and documented by clinicians within 24 h.

The UF treatment period was generally for 3 days. On the 4th Day, the effect of UF was observed, and on the 7th Day, the recovery of diuretic sensitivity evaluated. Thus, measurements for UF parameters were taken on Day 1, Day 2, Day 3, Day 4 and Day 7. In the UU group, the parameters of USCOM were also monitored on Day 1, Day 2, Day 3, Day 4 and Day 7 using a non-invasive USCOM device, which employed transaortic or transpulmonary Doppler flow tracing; the valve area was estimated using the patient’s height, with subsequent calculation of CO.

### 2.6 Variable Collection and Definitions

Baseline data from the patients after enrollment were recorded, including age, gender and height. Test results were collected, including BNP, the blood urea nitrogen to creatinine ratio, creatinine, C-reactive protein, estimated glomerular filtration rate, hemoglobin, jugular venous pressure, left ventricular ejection fraction, neutrophils percentage, procalcitonin and white blood cell counts.

ADHF is a clinical syndrome characterized by newly developed AHF or a worsening of the previously diagnosed chronic heart failure, accompanied by progressive fluid retention, resulting in an abrupt decrease in CO and systemic congestion. In addition, the definitions of comorbidities related to ADHF included atrial fibrillation, cerebral stroke, chronic obstructive pulmonary disease, diabetes mellitus, dilated cardiomyopathy, hypertension, hyperlipidemia, ischemic cardiomyopathy, renal insufficiency and valvular heart disease, in accordance with guidelines and previous literature reviews [26, 30].

The USCOM monitored parameters included the SV index (SVI), SV variation (SVV), CO, cardiac index, systemic vascular resistance (SVR), SVRI and the velocity time integral (VTI). CO refers to the total volume of blood ejected by one side of the heart per minute and is one of the most direct indicators reflecting cardiac function. CO was estimated by heart rate (HR) and the flow calculated from the VTI and the cross-sectional area of the valve orifice. VTI refers to the integral of blood flow velocity over a single ejection time. Cardiac index was calculated by dividing CO by the body surface area. FTc refers to the time required by the heart for systolic ejection, which was calculated using Bazett’s formula [17]. SV was calculated by measuring the Doppler flow in the aortic valve, which refers to the amount of blood ejected into the aorta during each systole. SVV was the percentage change in SV with each systole, that is, the percentage of the difference between the maximum and minimum SV values within a certain period of time compared to the average SV value during that period [31]. SVRI refers to the force exerted by peripheral blood vessels on the circulating blood [32].

### 2.7 Endpoints

The primary endpoint was differences in the hemodynamics of patients in the U and UU groups during UF, monitored at Day 1, Day 2, Day 3, Day 4 and Day 7. The secondary endpoints were the identification of one or more indicators on the USCOM that could predict the endpoint of UF, in which, thresholds of a 30% or 50% reduction in BNP relative to baseline were set as criteria for reaching the UF endpoint. Additionally, the economic benefits including treatment costs (such as UF related costs, hospitalization expenses, costs of blood concentrator, hemodialysis circuit and continuous renal replacement therapy), hospitalization duration and re-hospitalization rates at ≤30 days were also assessed between the two groups.

### 2.8 Statistical Analysis

All statistical analyses were conducted using SPSS (version 26.0, IBM Corp., Chicago, IL, USA) and *p*-values < 0.05 were deemed to be significant. Normality was tested using the Shapiro-Wilk test. The Kruskal-Wallis test was used to determine significant differences between groups when the data did not meet the assumptions for parametric tests, whereas a *t*-test or ANOVA are parametric tests based on the assumption that the data follow a normal distribution and exhibit homoscedasticity. Normally distributed data are presented as the mean ± SD. A Mann-Whitney U test or Wilcoxon rank sum test was used for continuous variables that were not normally distributed, and the results are reported as medians (Q1, Q3). To assess potential differences between categorical variables, a χ^2^ test or Fisher’s exact test were employed. A mixed linear model was utilized to analyze repeated measurement data to obtain the trend of hemodynamic indicators over time, and the Bonferroni correction was applied to perform statistical comparisons between the two groups at each time point. The change rate at each time point refers to the percentage change compared to the baseline (Day 1). Spearman’s correlation analysis was used to establish correlations between the changes of USCOM output and UF parameters on Day 4 compared to baseline UU treatments. Univariate and multivariate logistic regression analysis were employed to identify independent predictors, and receiver operating characteristic (ROC) curves and area under the curve (AUC) values to establish the prediction model. The fit of the model was assessed using the Hosmer-Lemeshow test. A regression imputation was employed to fill in the missing data in the present trial.

The sample size was not calculated prior to enrollment, but post hoc power estimates were carried out using G*Power 3.1 software (University of Dusseldorf, Dusseldorf, Germany). Based on previously published study by Liu *et al*. [33] and the primary endpoint of the present trial, the assessment of hemodynamics differences between the U and UU groups during UF was considered, with monitoring performed at Day 1, Day 2, Day 3, Day 4 and Day 7. The present trial particularly focused on the time to achieve UF endpoints during emergency hospitalization in patients with AHF, analyzing the correlation between hemodynamic changes in the UU group and the U endpoint markers before and after achieving U achievement. The assumption was made that the UU group would have a reduced average length of emergency stay of 8 days compared to conventional medication, and the U group time by 4.5 days. Under the premise of a one-sided *p* < 0.05, an 80% power for a statistically significant difference to achieve an 80% reduction in emergency stay length for the UU group compared to the U group was required. The effect size of 80% was considered to be a reasonable estimate in the present trial. A total of 40 patients (20 per group) were enrolled for the final analysis, with an average reduction in emergency stay length of 7.7 days for the UU group (n = 20) and 4.2 days for the U group (n = 20), with an effect size of 80%. The power of this study was calculated to be 79.9% under a one-sided test at α = 0.05 and 70% under two-sided test at α = 0.05.

## 3. Results

### 3.1 Baseline and Clinical Characteristics of Patients

In this trial, the overall population exhibited the following median (interquartile range (IQR)) values: hemoglobin concentrations of 117.0 g/L (110.0, 124.8 g/L), creatinine 128.0 µmoI/L (86.3, 163.7 µmoI/L), an estimated glomerular filtration rate of 48.9 mL/min/1.73 m^2^ (29.5, 64.4 mL/min/1.73 m^2^), left ventricular ejection fraction 44.0% (32.9, 56.8%) and a BNP concentration of 2098.8 ng/L (922.0, 3929.0 ng/L). Notably, 95% of patients presented with renal insufficiency, 72.5% hypertension and 65.0% were diagnosed with diabetes mellitus. Cerebral stroke was diagnosed in 22.5% of cases, while chronic obstructive pulmonary disease occurred in 1 case. Additionally, the congestion status of the two groups showed that the blood urea nitrogen to creatinine ratio was 26.6 in the UU group and 24.6 for the U group. Among 28 patients (70.0%) received positive inotropic support medication, mainly dopamine (47.5%), followed by a type 3 phosphodiesterase inhibitor (milrinone) (32.5%) in both groups. It is worth noting that the baseline and clinical characteristics of the two treatment groups were closely matched (all *p* > 0.05; Table 1).

**Table 1.** **Baseline and clinical characteristics of the trial patients**.

		Total (n = 40)	UU Group (n = 20)	U group (n = 20)	*p*-value
Gender, n (%)					
	Male	21 (52.5)	13 (65.0)	8 (40.0)	0.205
	Female	19 (47.5)	7 (35.0)	12 (60.0)	
Age (years)		74.5 (65.3, 82.0)	79.0 (69.5, 83.8)	72.0 (57.8, 81.5)	0.093
Height (cm)		164.5 (160.0, 171.0)	164.5 (159.3, 171.5)	163.5 (160.0, 171.0)	0.888
Weight (kg)		69.0 (60.0, 80.0)	63.5 (57.8, 75.0)	71.0 (64.3, 83.8)	0.157
BMI (kg/m^2^)		24.8 (22.6, 29.0)	24.4 (21.9, 27.9)	26.2 (22.8, 30.7)	0.303
Hemoglobin (g/L)		117.0 (110.0, 124.8)	119.0 (107.3, 128.8)	116.0 (110.0, 123.0)	0.369
Creatinine (µmoI/L)		128.0 (86.3, 163.7)	96.1 (85.2, 135.8)	147.2 (89.0, 176.0)	0.142
Estimated glomerular filtration rate (mL/min/1.73 m^2^)		48.9 (29.5, 64.4)	49.8 (41.6, 61.3)	35.3 (25.7, 70.0)	0.142
C-reactive protein (mg/L)		7.2 (5.8, 10.3)	7.2 (4.5, 7.5)	8.2 (6.2, 12.2)	0.083
Procalcitonin (ng/mL)		1.5 (0.2, 2.0)	1.3 (0.1, 1.8)	1.5 (0.9, 2.8)	0.316
White blood cell count (×10^9^/L)		5.6 (5.1, 6.4)	5.5 (5.0, 5.9)	5.7 (5.2, 6.8)	0.203
Neutrophil (%)		70.6 (68.2, 75.7)	70.2 (66.4, 75.7)	71.4 (68.8, 76.7)	0.208
Left ventricular ejection fraction (%)		44.0 (32.9, 56.8)	46.5 (33.0, 56.8)	41.7 (32.9, 57.5)	0.947
BNP (pg/mL)		2098.8 (922.0, 3929.0)	1990.3 (1080.0, 4562.2)	2098.8 (703.0, 3506.6)	0.242
Jugular venous pressure (cmH_2_O)		18.0 (16.0, 19.8)	18.0 (16.0, 19.7)	18.5 (13.8, 19.8)	0.902
Blood urea nitrogen to creatinine ratio		25.3 (21.6, 29.4)	26.6 (21.8, 30.4)	24.6 (21.3, 27.3)	0.337
Etiology of ADHF, n (%)					
	Ischemic cardiomyopathy	21 (52.5)	10 (50.0)	11 (55.0)	0.083
	Dilated cardiomyopathy	7 (17.5)	4 (20.0)	3 (15.0)	0.316
	Valvular heart disease	3 (33.3)	2 (10.0)	1 (5.0)	0.203
	Atrial fibrillation	19 (47.5)	10 (50.0)	9 (45.0)	0.208
	Hypertension	29 (72.5)	13 (65.0)	16 (80.0)	0.478
	Hyperlipidemia	10 (25.0)	4 (20.0)	6 (30.0)	0.472
	Chronic obstructive pulmonary disease	1 (2.5)	1 (5.0)	0 (0.0)	1.000
	Renal insufficiency	38 (95.0)	20 (100.0)	18 (90.0)	0.490
	Diabetes mellitus	26 (65.0)	12 (60.0)	14 (70.0)	0.512
	Cerebral stroke	9 (22.5)	7 (35.0)	2 (10.0)	0.132
Positive inotropic agents, n (%)		28 (70.0)	14 (70.0)	13 (65)	1.000
	Dopamine	19 (47.5)	10 (50.0)	9 (45.0)	1.000
	Type 3 phosphodiesterase inhibitor (milrinone)	13 (32.5)	4 (20.0)	9 (45.0)	0.176
	Calcium sensitizer (levosimendan)	7 (17.5)	4 (20.0)	3 (15.0)	1.000
	Epinephrine	3 (7.5)	1 (5.0)	2 (10.0)	1.000
	Hydroxylamine	3 (7.5)	2 (10.0)	1 (5.0)	1.000
	Isoproterenol	2 (5.0)	1 (5.0)	1 (5.0)	1.000
	Digitalis medication	11 (27.5)	4 (20.0)	7 (35.0)	0.480

Note. Data are presented as medians (Q1, Q3) and n (%). Abbreviations: ADHF, acute decompensated heart failure; BMI, body mass index; BNP, B-type natriuretic peptide; U group, ultrafiltration group; UU group, ultrafiltration + ultrasonic cardiac output monitor group.

### 3.2 Primary Endpoint

**Fig. 1.**
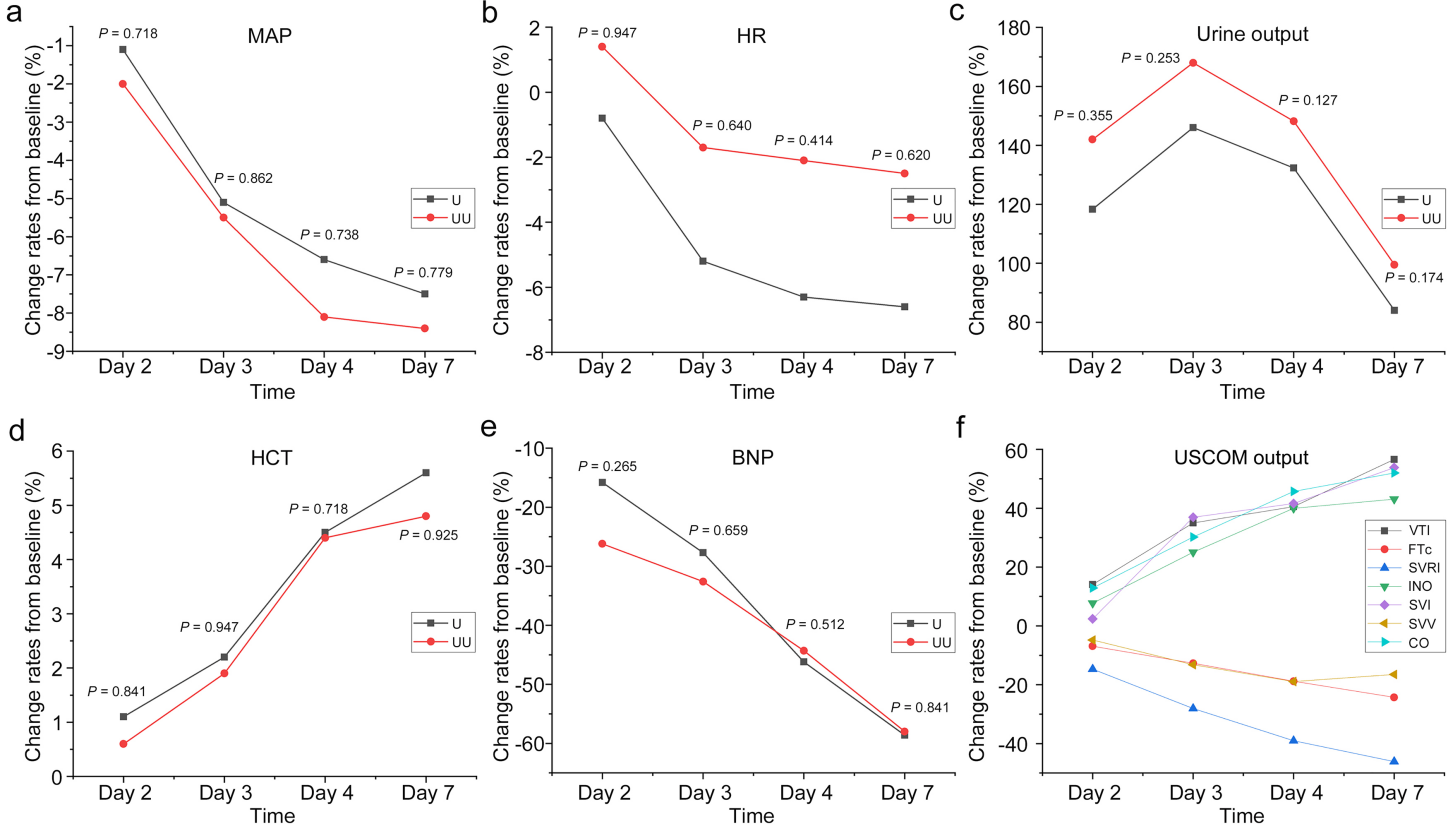
**The trends of change rates on Day 2, Day 3, Day 4 and Day 7 from baseline in (a) MAP, (b) HR, (c) urine output, (d) HCT and (e) BNP measured by UF parameters in the U and UU groups, and (f) USCOM output in the UU group**. BNP, B-type natriuretic peptide; CO, cardiac output; FTc, corrected flow time; HCT, hematocrit; HR, heart rate; INO, inotropy; MAP, mean arterial pressure; SVI, stroke volume index; SVV, stroke volume variation; SVRI, systemic vascular resistance index; VTI, velocity time integral; UF, ultrafiltration; USCOM, ultrasonic cardiac output monitor; U group, ultrafiltration group; UU group, ultrafiltration + ultrasonic cardiac output monitor group.

As shown in Fig. 1, there were no statistically significant differences between the two groups (UU and U) in the change rates of variables such as mean arterial pressure (MAP), HR, urine output, HCT and BNP at different time points (all *p* > 0.05). Specifically, the change rates of MAP and BNP showed a linear decrease with increasing UF time, while HCT gradually increased as UF progressed. According to the hemodynamic indicators shown by USCOM, FTc, SV and SVRI significantly decreased with the extension of UF time, whereas VTI, INO, CO and SVI significantly increased. Except for urine output and BNP, which exhibited a sharp decrease in the change rate after Day 4, other variables stabilized between Day 4 and Day 7. These findings suggest that changes in hemodynamic indicators may help predict the likelihood of achieving UF targets.

Similarly, from Table 2, we can more clearly perceive the indicators related to UF. In both the U and UU groups, diastolic blood pressure, urine output, HCT and BNP exhibited a linear increase or decrease in the changes from baseline as UF progressed (*p*-trend ≤ 0.05). Additionally, compared to Day 4, only the change rate of urine output and BNP showed significant differences on Day 7 (*p* < 0.05), while the change rates of other indicators stabilized after Day 4. However, regarding the change rates in USCOM parameters, except for SVV, the change rates of other hemodynamic indicators showed a linear increase or decrease as UF progressed (all *p* < 0.05). When comparing the changes from baseline on Day 4 and Day 7, significant differences were still observed in the change rates of CO, cardiac index, INO, SVRI, SVR, FTc and VTI (Table 2). These findings raise the question of whether it is possible to combine hemodynamic indicators that show a linear change over the course of UF, along with those that stabilize by Day 4 and Day 7, to perform a correlation analysis and predict the optimal time for UF achievement in patients with AHF.

**Table 2.** **Changes in hemodynamic indices measured by UF parameters at five different time points compared to baseline in the U and UU groups and USCOM output in the UU group**.

		Day 1	△Day 2	△Day 3	△Day 4	△Day 7	*p*-value	*p-*value	*p-*value
							(trend for 1–4 days)	(Day 7 vs. Day 4)	(trend)
UF parameters of the U group	SBP (mmHg)	133.5 (117.3, 154.5)	–1.0 (–11.5, 5.8)	–3.5 (–14.8, 5.0)	0.0 (–14.8, 7.0)	–6.0 (–15.2, 2.0)	0.072	0.621	0.065
	DBP (mmHg)	70.0 (64.0, 81.3)	–2.0 (–8.2, 5.2)	–5.5 (–14.0, 1.5)	–8.0 (–13.2, 0.2)	–8.0 (–16.2, 0.5)	0.003	0.726	0.007
	MAP (mmHg)	94.8 (83.3, 104.5)	–0.5 (–6.1, 3.5)	–5.7 (–14.2, 2.7)	–6.2 (–16.0, 1.0)	–8.3 (–14.0, 0.4)	0.004	0.634	0.006
	HR (bpm)	82.0 (72.0, 91.8)	3.5 (–6.2, 5.2)	–3.5 (–11.2, 3.8)	–7.0 (–14.2, 2.5)	–8.0 (–15.0, 2.2)	0.194	0.649	0.094
	Urine output (mL)	950.0 (700.0, 1362.5)	750.0 (562.5, 1195.0)	1175.0 (637.5, 1625.0)	850.0 (568.8, 1350.0)	475.0 (237.5, 925.0)	0.726	<0.001	0.005
	SpO_2_ (%)	96.0 (95.0, 97.0)	0.5 (0.0, 1.2)	0.5 (0.0, 2.0)	1.0 (0.0, 2.0)	1.0 (0.0, 2.0)	0.266	0.359	0.053
	HCT (%)	35.0 (33.1, 36.3)	–0.0 (–1.1, 2.0)	1.0 (–0.5, 2.4)	2.4 (0.1, 3.4)	1.2 (0.7, 3.2)	0.007	0.431	0.001
	BNP (pg/mL)	2098.9 (709.7, 3015.8)	–168.4 (–844.8, –29.8)	–570.2 (–1276.9, –129.2)	–1218.1 (–1646.0, –328.7)	–1512.0 (–1891.5, –406.4)	<0.001	0.007	<0.001
UF parameters of the UU group	SBP (mmHg)	128.5 (111.2, 148.0)	–2.0 (–7.0, 2.5)	–8.5 (–17.5, 1.2)	–8.5 (–17.8, –3.8)	–9.5 (–17.0, –2.8)	0.070	0.492	0.056
	DBP (mmHg)	66.5 (63.8, 81.0)	0.0 (–4.2, 2.0)	–3.0 (–9.8, 1.0)	–6.0 (–12.5, –2.0)	–5.5 (–14.2, –2.0)	0.004	0.911	0.021
	MAP (mmHg)	90.2 (81.0, 98.7)	–0.7 (–5.3, 1.7)	–5.2 (–10.2, –0.4)	–6.5 (–12.8, –4.5)	–5.2 (–11.9, –2.3)	0.004	0.720	0.011
	HR (bpm)	84.5 (71.8, 106.2)	0.5 (–13.0, 13.0)	–2.7 (–9.9, 10.6)	–3.0 (–13.2, 13.5)	–3.0 (–14.5, 9.8)	0.400	0.755	0.331
	Urine output (mL)	895.0 (700.0, 1000.0)	1205.0 (836.0, 1612.5)	1375.0 (961.0, 2000.0)	1139.0 (975.0, 1462.5)	750.0 (500.0, 987.5)	0.857	<0.001	<0.001
	SpO_2_ (%)	96.0 (95.0, 97.0)	1.0 (–1.0, 2.2)	1.0 (0.0, 2.0)	1.0 (1.0, 2.0)	1.5 (0.0, 3.0)	0.237	0.644	0.104
	HCT (%)	35.9 (33.6, 38.1)	0.1 (–0.7, 1.5)	1.2 (–0.1, 1.9)	1.9 (0.5, 2.6)	2.2 (0.1, 3.0)	0.002	0.466	0.001
	BNP (pg/mL)	1647.0 (1047.0, 4041.3)	–314.0 (–794.6, –204.4)	–748.8 (–1221.4, –248.5)	–837.0 (–1691.0, –352.7)	–1036.5 (–1853.2, –502.9)	0.002	<0.001	<0.001
USCOM monitoring output of the UU group	SV (mL)	22.3 (14.1, 33.5)	0.7 (–3.5, 6.2)	9.2 (1.5, 14.9)	8.9 (–0.9, 21.2)	12.1 (3.6, 21.9)	<0.001	0.279	<0.001
	SVI (mL/beats)	12.5 (7.8, 18.8)	0.4 (–2.0, 3.2)	4.6 (0.8, 7.3)	5.1 (–0.5, 11.0)	7.0 (2.0, 11.7)	<0.001	0.250	<0.001
	SVV (%)	58.5 (34.5, 72.5)	–3.5 (–16.5, 16.0)	0.2 (–28.8, 14.0)	–5.0 (–30.0, 14.2)	–9.5 (–27.8, 16.0)	0.430	0.505	0.927
	CO (L/min)	1.9 (1.6, 2.3)	0.3 (–0.2, 0.4)	0.5 (0.3, 1.0)	0.8 (0.3, 1.1)	1.2 (0.2, 1.6)	<0.001	0.027	<0.001
	Cardiac index, (L/min/m^2^)	1.1 (0.8, 1.3)	0.1 (–0.1, 0.2)	0.3 (0.2, 0.5)	0.4 (0.2, 0.7)	0.6 (0.1, 0.8)	<0.001	0.020	<0.001
	INO (W/m^2^)	0.6 (0.4, 0.9)	0.1 (0.0, 0.2)	0.1 (0.1, 0.3)	0.3 (0.2, 0.4)	0.3 (0.2, 0.4)	<0.001	0.009	<0.001
	SVRI (mmHg·min/mL)	8339 (6356, 13,527)	–1116 (–1975.5, –689.5)	–2011.5 (–3870.0, –1565.8)	–3594.5 (–4674.0, –2180.0)	–4080 (–6530.0, –2656.0)	<0.001	<0.001	<0.001
	SVR (mmHg·min/L)	4430 (3258, 7862)	–594 (–1130.0, –377.6)	–1106.1 (–2135.3, –863.9)	–1696.4 (–2779.5, –1188.6)	–1925.5 (–3745.3, –1282.1)	<0.001	<0.001	<0.001
	FTc (ms)	427 (388, 464)	–25 (–39.5, –8.5)	–46.5 (–65.2, –33.5)	–70.5 (–115.0, –54.8)	–103.5 (–144.2, –84.2)	<0.001	<0.001	<0.001
	VTI (cm)	8.1 (6.4, 9.4)	0.9 (0.4, 1.8)	2.6 (1.1, 4.1)	3.1 (1.8, 5.7)	5.3 (2.8, 7.1)	<0.001	<0.001	<0.001

Note. Data are presented as median (Q1, Q3) unless otherwise indicated. △ denotes changes from baseline. Abbreviations: BNP, B-type natriuretic peptide; CO, cardiac output; DBP, diastolic blood pressure; FTc, corrected flow time; HCT, hematocrit; HR, heart rate; INO, inotropy; MAP, mean arterial pressure; SBP, systolic blood pressure; SpO_2_, oxygen saturation; SV, stroke volume; SVI, stroke volume index; SVR, systemic vascular resistance; SVRI, systemic vascular resistance index; SVV, stroke volume variation; UF, ultrafiltration; USCOM, ultrasonic cardiac output monitor; U group, ultrafiltration group; UU group, ultrafiltration + ultrasonic cardiac output monitor group; VTI, velocity time integral.

### 3.3 Secondary Endpoints

#### 3.3.1 Correlations of the Changes Rates From Baseline on Day 4 Between USCOM Output and UF Parameters 

We conducted an analysis using **Supplementary Table 1**, and the results showed that change rates in SV and SVI in the USCOM were negatively correlated with change rates in MAP (r = –0.329, *p* = 0.003), and positively correlated with change rates of HR (r = 0.673, *p* < 0.001) and SpO_2_ (r = 0.229, *p* = 0.041) during the UF process (**Supplementary Table 2**). The change rate of SVV in USCOM was positively correlated with the change rate of MAP (r = 0.296, *p* = 0.008), while the change rate of INO in USCOM was negatively correlated with the BNP change rate (r = –0.473, *p* < 0.001). The change rates of SVR and SVRI in USCOM were positively correlated with the change rates of MAP (r = 0.322, *p* = 0.004), HCT (r = 0.251, *p* = 0.012) and BNP (r = 0.422, *p* < 0.001) during the UF process. The FTc change rate in USCOM was positively correlated with the change rates of MAP (r = 0.280, *p* = 0.012), urine output (r = 0.255, *p* = 0.022) and BNP (r = 0.353, *p* = 0.001), and negatively correlated with the change rates of SpO_2_ (r = –0.198, *p* = 0.048) and HCT (r = –0.500, *p* < 0.001) during the UF process. The VTI change rate in USCOM was positively correlated with the SpO_2_ change rate (r = 0.197, *p* = 0.050) and negatively correlated with the change rates of MAP (r = –0.263, *p* = 0.019), HR (r = –0.454, *p* < 0.001), urine output (r = –0.27, *p* = 0.016) and BNP (r = –0.602, *p* < 0.001) during the UF process. The HR change rate was positively correlated with the change rates of MAP (r = 0.273, *p* = 0.014) and BNP (r = 0.229, *p* = 0.041), and negatively correlated with the SpO_2_ change rate (r = –0.222, *p* = 0.048) (**Supplementary Table 2**). Therefore, indicators such as SV, SVI, SVV, INO, SVR, SVRI, FTc, VTI and HR change rates in USCOM were correlated with UF indicators.

#### 3.3.2 Independent Predictors of 30% or 50% Decrease in BNP Relative to Baseline

Before performing the multivariate regression analysis, we selected variables based not only on their statistical significance in univariate analysis (*p* < 0.05, as shown in Table 2) but also on their clinical relevance. Additionally, we incorporated clinical experience to select variables for univariate regression analysis, which are listed in Table 3, including: age (years), SVI (mL/beats), SVV (%), CO (L/min), INO (W/m^2^), cardiac index (L/min/m^2^), SVRI (mmHg⋅min/mL), FTc (ms), VTI (cm), HR (bpm), SpO_2_ (%), HCT (%), urine output (mL) and MAP (mmHg). Based on these values, we included the variables with *p*-values < 0.05 from the univariate regression analysis (SVI, INO, SVRI, FTc, VTI) into the multivariate regression analysis to identify variables with a significant impact on achieving UF endpoints.

**Table 3.** **Correlation of standard and substandard UF endpoints and USCOM output based on a 30% reduction on Day 4 compared to baseline in BNP from the baseline during UF**.

	Univariate analysis			Multivariate analysis				Hosmer-Lemeshow test
	Substandard (BNP)	Standard (BNP)	*p*-value	Substandard (BNP)	Standard (BNP)	OR (95% CI)	*p*-value	
Age (years)	79.0 (72.0, 84.0)	78.0 (68.8, 83.0)	0.247	79.0 (72.0, 84.0)	78.0 (68.8, 83.0)		0.549	
SVI (mL/beats)	22.5 (–6.6, 47.8)	43.9 (1.3, 89.7)	0.033	22.5 (–6.6, 47.8)	43.9 (1.3, 89.7)		0.162	
SVV (%)	–1.3 (–29.9, 37.6)	–17.4 (–40.9, 39.5)	0.358	–1.3 (–29.9, 37.6)	–17.4 (–40.9, 39.5)		0.933	
Cardiac output (L/min)	19.8 (5.8, 47.9)	34.1 (9.2, 73.6)	0.145	19.8 (5.8, 47.9)	34.1 (9.2, 73.6)		0.574	
INO (W/m^2^)	13.5 (4.2, 30.5)	39.5 (26.7, 63.9)	<0.001	13.5 (4.2, 30.5)	39.5 (26.7, 63.9)	1.028 (1.005–1.051)	0.015	0.814
Cardiac index (L/min/m^2^)	19.8 (5.8, 47.9)	34.1 (9.2, 73.6)	0.145	19.8 (5.8, 47.9)	34.1 (9.2, 73.6)		0.574	
SVRI (mmHg·min/mL)	–21.0 (–33.8, –14.2)	–38.3 (–48.8, –28.4)	<0.001	–21.0 (–33.8, –14.2)	–38.3 (–48.8, –28.4)	0.933 (0.892–0.976)	0.003	0.814
FTc (ms)	–10.0 (–13.7, –4.3)	–17.7 (–27.0, –9.2)	0.002	–10.0 (–13.7, –4.3)	–17.7 (–27.0, –9.2)		0.230	
VTI (cm)	18.1 (8.4, 38.1)	34.6 (22.4, 67.7)	0.003	18.1 (8.4, 38.1)	34.6 (22.4, 67.7)		0.126	
HR (bpm)	–0.8 (–10.5, 24.6)	–4.6 (–22.8, 14.0)	0.090	–0.8 (–10.5, 24.6)	–4.6 (–22.8, 14.0)		0.116	
SpO_2_ (%)	1.0 (–1.0, 2.1)	1.0 (0, 2.4)	0.128	1.0 (–1.0, 2.1)	1.0 (0, 2.4)		0.109	
HCT (%)	2.6 (–1.2, 5.5)	5.3 (–2.5, 7.9)	0.291	2.6 (–1.2, 5.5)	5.3 (–2.5, 7.9)		0.794	
Urine output (mL)	133.8 (98.6, 200.0)	124.9 (89.3, 184.0)	0.306	133.8 (98.6, 200.0)	124.9 (89.3, 184.0)		0.735	
MAP (mmHg)	–4.0 (–10.9, –0.9)	–6.5 (–11.5, –1.7)	0.390	–4.0 (–10.9, –0.9)	–6.5 (–11.5, –1.7)		0.458	

Note. Data represent the change rates, except the age and are expressed as median (Q1, Q3). Abbreviations: BNP, B-type natriuretic peptide; CI, confidence interval; FTc, corrected flow time; HCT, hematocrit; HR, heart rate; INO, inotropy; MAP, mean arterial pressure; OR, odds ratio; SpO_2_, oxygen saturation; SVI, stroke volume index; SVRI, systemic vascular resistance index; SVV, stroke volume variation; UF, ultrafiltration; USCOM, ultrasonic cardiac output monitor; VTI, velocity time integral.

Since a 30% or 50% decrease in BNP is the clinical standard for achieving UF, we used these two thresholds as cut-off values to separate the study patients into those who met the standard and those who did not on Day 4 compared to the baseline. Multivariate logistic regression showed that INO (odds ratio (OR) 1.028, 95% confidence interval (CI): 1.005–1.051; *p* = 0.015) and SVRI (OR 0.933, 95% CI: 0.892–0.976; *p* = 0.003) on USCOM were found to be factors correlated to a 30% reduction in BNP on Day 4 compared to the baseline (Table 3), while FTc (OR 0.916, 95% CI: 0.865–0.969; *p* = 0.002) and HR (OR 0.965, 95% CI: 0.939–0.991; *p* = 0.009) on USCOM were found to be independent factors correlated to a 50% reduction in BNP on Day 4 compared to the baseline (Table 4).

**Table 4.** **Correlations between standard and substandard UF endpoints and USCOM output based on a 50% reduction on Day 4 compared to baseline in BNP from the baseline during UF**.

	Univariate analysis			Multivariate analysis				Hosmer-Lemeshow test
	Substandard (BNP)	Standard (BNP)	*p*-value	Substandard (BNP)	Standard (BNP)	OR (95% CI)	*p*-value	
Age (years)	79.0 (73.0, 84.0)	71.0 (63.0, 79.0)	0.001	79.0 (73.0, 84.0)	71.0 (63.0, 79.0)		0.065	
SVI (mL/beats)	26.3 (–4.2, 56.3)	52.6 (20.8, 173.5)	0.021	26.3 (–4.2, 56.3)	52.6 (20.8, 173.5)		0.564	
SVV (%)	–3.7 (–34.6, 37.4)	–18.2 (–39.8, 39.4)	0.617	–3.7 (–34.6, 37.4)	–18.2 (–39.8, 39.4)		0.415	
CO (L/min)	26.0 (8.6, 56.4)	31.3 (12.4, 90.5)	0.395	26.0 (8.6, 56.4)	31.3 (12.4, 90.5)		0.441	
INO (W/m^2^)	25.0 (7.7, 45.1)	39.8 (28.4, 92.1)	0.006	25.0 (7.7, 45.1)	39.8 (28.4, 92.1)		0.089	
Cardiac index (L/min/m^2^)	26.0 (8.6, 56.4)	31.3 (12.4, 90.5)	0.395	26.0 (8.6, 56.4)	31.3 (12.4, 90.5)		0.441	
SVRI (mmHg·min/mL)	–29.0 (–38.0, –18.3)	–39.9 (–48.9, –31.6)	0.006	–29.0 (–38.0, –18.3)	–39.9 (–48.9, –31.6)		0.136	
FTc (ms)	–10.6 (–19.8, –5.5)	–23.3 (–31.3, –13.3)	0.001	–10.6 (–19.8, –5.5)	–23.3 (–31.3, –13.3)	0.916 (0.865–0.969)	0.002	0.655
VTI (cm)	26.4 (11.8, 46.7)	55.7 (29.7, 78.9)	0.002	26.4 (11.8, 46.7)	55.7 (29.7, 78.9)		0.560	
HR (bpm)	2.8 (–11.0, 21.4)	–14.1 (–34.9, 0)	0.002	2.8 (–11.0, 21.4)	–14.1 (–34.9, 0)	0.965 (0.939–0.991)	0.009	0.655
SpO_2_ (%)	1.0 (0, 2.1)	2.0 (0, 3.2)	0.522	1.0 (0, 2.1)	2.0 (0, 3.2)		0.367	
HCT (%)	4.3 (–0.5, 6.7)	1.9 (–4.2, 8.5)	0.359	4.3 (–0.5, 6.7)	1.9 (–4.2, 8.5)		0.182	
Urine output (mL)	138.5 (101.1, 193.1)	106.5 (78.6, 134.3)	0.005	138.5 (101.1, 193.1)	106.5 (78.6, 134.3)		0.094	
MAP (mmHg)	–5.2 (–13.1, –0.3)	–6.6 (–10.8, –2.4)	0.733	–5.2 (–13.1, –0.3)	–6.6 (–10.8, –2.4)		0.326	

Note. Data represent the change rates, except the age, and are expressed as median (Q1, Q3). Abbreviations: BNP, B-type natriuretic peptide; CI, confidence interval; CO, cardiac output; FTc, corrected flow time; HCT, hematocrit; HR, heart rate; INO, inotropy; MAP, mean arterial pressure; OR, odds ratio; SpO_2_, oxygen saturation; SVI, stroke volume index; SVRI, systemic vascular resistance index; SVV, stroke volume variation; UF, ultrafiltration; USCOM, ultrasonic cardiac output monitor; VTI, velocity time integral.

Additionally, in this trial, using a 30% reduction in BNP as the criterion for UF success, the proportion of patients achieving the standard by Day 7 was 90% in the U group and 100% in the UU group, with a median achievement time of 4.0 days for both groups. The average time to standard achievement was 5.9 days in the UU group and 6.0 days in the U group.

By Day 7, using a 50% reduction in BNP as the criterion for UF success, the proportion of patients achieving the standard was 70% in both the U and UU groups, with a median achievement time of 7 days. The average time to standard achievement was 3.5 days in the UU group and 3.9 days in the U group.

#### 3.3.3 Predictive Model of USCOM Parameters for Achieving UF Standards

After including variables directly affecting UF standards (all *p* < 0.05) in multiple regression analysis, we focused on the variables included from univariate analysis and performed ROC curve analysis for these variables in single or combined situations to predict the possibility model of achieving UF attainments.

**Fig. 2.**
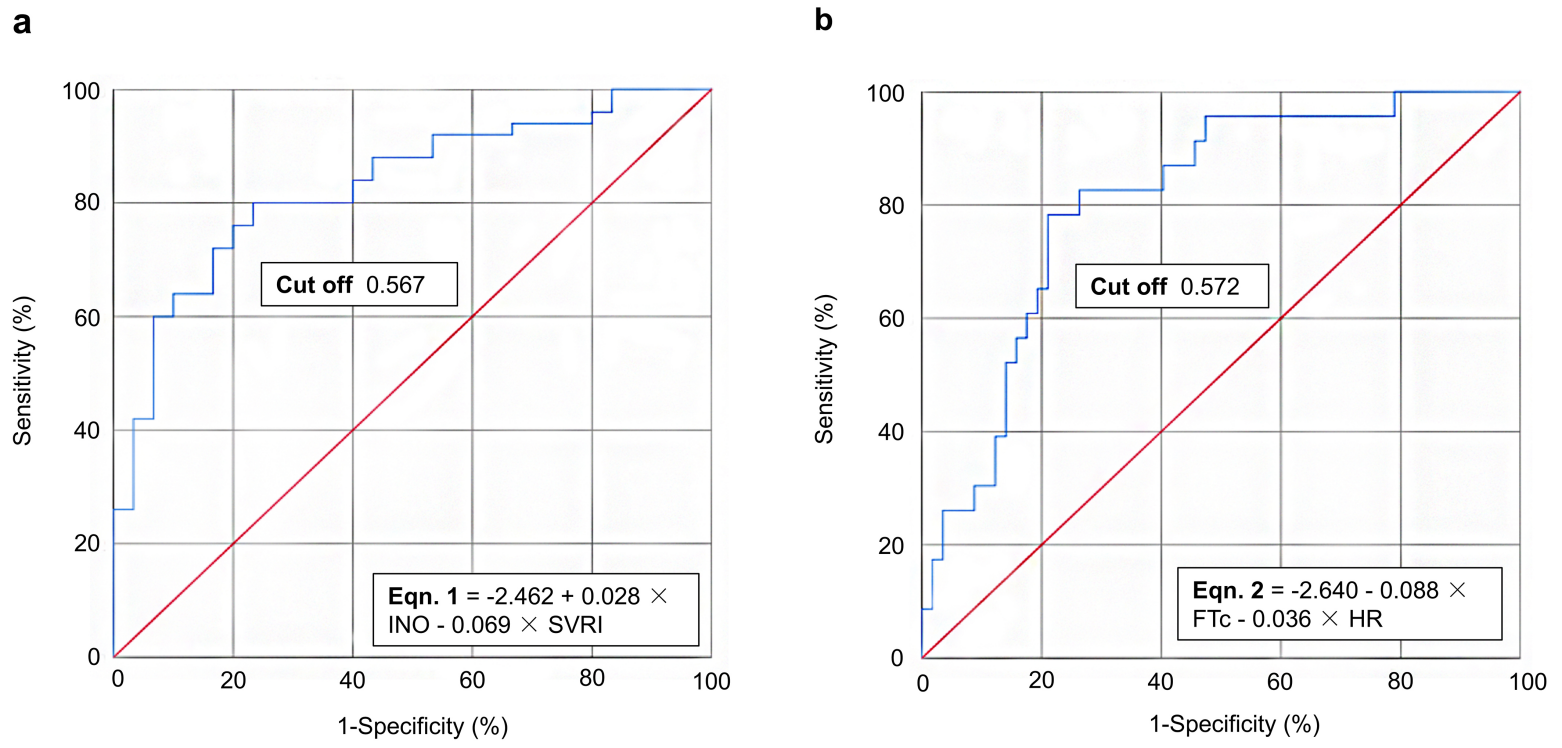
**ROC curve analysis for achieving UF standards of thresholds of (a) 30% and (b) 50% reduction in BNP concentrations relative to baseline on Day 4**. BNP, B-type natriuretic peptide; FTc, corrected flow time; HR, heart rate; INO, inotropy; ROC, receiver operating characteristic; SVRI, systemic vascular resistance index; UF, ultrafiltration.

After including variables directly affecting UF standards (all *p* < 0.05) in multiple regression analysis, we focused on the influential INO and SVRI variables and performed ROC curve analysis for these variables in single or combined situations to predict the possibility model of achieving UF attainments. By evaluating the sensitivity (true positive rate), specificity (true negative rate), and accuracy, we found that considering a reduction in BNP on Day 4 compared to a baseline >30%, Eqn. 1 for determining UF endpoints was: –2.462 + 0.028 × INO – 0.069 × SVRI (AUC ROC 0.831, 95% CI: 0.741–0.920; Hosmer-Lemeshow index 0.814), with a predicted sensitivity, specificity and accuracy of 70% (37%, 89%), 83% (47%, 97%) and 75.0%, with a cut-off value of 0.567 (Fig. 2a and** Supplementary Table 3**). In other words, when using USCOM for UF monitoring, we could predict UF attainment using this formula thus avoiding over UF for patients.

For the condition of considering a reduction in BNP on Day 4 compared to a baseline of >50%, Eqn. 2 was: –2.640 – 0.088 × FTc – 0.036 × HR (AUC ROC 0.809, 95% CI: 0.709–0.909; Hosmer-Lemeshow index 0.655), with a predicted cut-off value of 0.572 (Fig. 2b and **Supplementary Table 4**). This means that, according to the 50% reduction in the BNP standard, when inserting the corresponding USCOM variables, if the probability value (*p*) was >0.572, it indicated that UF attainments had been achieved with sensitivity, specificity and accuracy of this model of 83% (52%, 96%), 63% (33%, 86%) and 72.5%, respectively.

### 3.4 Adverse Events 

No adverse events were reported due to USCOM within 24 h, indicating that USCOM was a safe and non-invasive monitoring device.

### 3.5 Economic Benefits After Using USCOM + UF

The use of USCOM could significantly reduce treatment costs including UF related costs (1309.9 vs. 955.8 USD, *p* = 0.030), hospitalization expenses (5175.5 vs. 3524.6 USD, *p* = 0.007) and costs of blood concentrator (504.0 vs. 336.0 USD, *p* = 0.046) and the hemodialysis circuit (546.0 vs. 364.0 USD, *p* = 0.046), as well as a shorter mean hospitalization duration (9.3 vs. 12.8 days, *p* = 0.015). In addition, there was no significant difference in re-hospitalization rates associated with heart failure at ≤30 days (25.0% vs. 20.0%, *p* = 0.705) and costs related to continuous renal replacement therapy (268.8.0 vs. 156.8.0 USD, *p* = 0.142) between the U and UU groups (Table 5).

**Table 5.** **Economic benefits after using USCOM + UF**.

		Total (n = 40)	UU group (n = 20)	U group (n = 20)	*p*-value
Treatment costs (USD)					
	UF related costs	1208.8 (896.1, 1327.0)	955.8 (862.4, 1277.4)	1309.9 (919.8, 1331.0)	0.030
	Hospitalization expenses	3769.5 (3152.8, 5506.1)	3524.6 (2923.1, 4245.4)	5175.5 (3465.1, 7732.1)	0.007
	Blood concentrator	420.0 (336.0, 504.0)	336.0 (336.0, 504.0)	504.0 (336.0, 504.0)	0.046
	Hemodialysis circuit	455.0 (364.0, 546.0)	364.0 (364.0, 546.0)	546.0 (364.0, 546.0)	0.046
	Continuous renal replacement therapy	235.2 (156.8, 268.8)	156.8 (156.8, 268.8)	268.8 (156.8, 268.8)	0.142
Hospitalization durations (Days)		11.0 ± 4.5	9.3 ± 2.5	12.8 ± 5.4	0.015
Re-hospitalization at 30 days or less, n (%)		9 (22.5)	4 (20.0)	5 (25.0)	0.705

Note. Data are presented as medians (Q1, Q3) and the mean ± SD unless otherwise indicated. Abbreviations: UF, ultrafiltration; USCOM, ultrasonic cardiac output monitor; U group, ultrafiltration group; UU group, ultrafiltration + ultrasonic cardiac output monitor group.

## 4. Discussion

The present study found that change rates in MAP, HR, urine output, HCT and BNP over 7 days were similar between the U and UU groups. Our study also evaluated whether USCOM could be used to estimate the endpoints of UF for ADHF patients and provided two UF-endpoint prediction formulae for evaluating a 30% reduction in BNP: –2.462 + 0.028 × INO – 0.069 × SVRI, and for a 50% reduction of BNP: –2.640 – 0.088 × FTc – 0.036 × HR. Moreover, UF combined with USCOM also reduced the financial burden of treatment and hospitalization for patients.

The introduction of non-invasive devices for monitoring CO in AHF patients represents a significant advance in that they can reduce the occurrence of complications (e.g., infection, thrombosis) compared with invasive hemodynamic monitoring [34]. The present trial also confirmed that USCOM was a safe non-invasive device, as no adverse events due to USCOM were observed within 24 h. Current non-invasive approaches for determining CO include impedance cardiography and echocardiography, but other techniques are under investigation but are proving to have varying efficacies [35]. Operator dependence was undoubtedly the greatest limitation for the application of echocardiography. That is, the operator required advanced experience and echocardiography training, so that the learning curve was even longer compared to that of other non-invasive hemodynamic monitoring approaches [36]. Impedance cardiography was an operator-independent cost-effective and non-invasive approach, but the measurement accuracy might be limited to pathological states, such as too low or high CO values, valvular regurgitation, intracardiac shunts and the incidence of arrhythmia [37]. The clinical application worth of USCOM remains controversial, with its accuracy and precision being assessed with varied results compared with other non-invasive methods [38, 39]. USCOM was easy to operate so that trainees could reach the same level as the trainers after 50 operations, thus the learning curve for skill acquisition was significantly shorter [21]. Moreover, the addition of USCOM also significantly resulted in treatment cost savings and a reduced hospitalization stay length, which confirmed the cost-effectiveness of USCOM as previously reported [16]. Notably, USCOM was also susceptible to operational influences. Consequently, all patients in the UU group in the present trial were monitored by the same skilled operator and three consecutive measurements were made with a deviation of no more than 10% each time, in order to ensure the consistency and reliability of the data.

In the present trial, there were clear differences in the changes of hemodynamic parameters during UF, which reached a steady-state level after 4 days. Since BNP was the standard biomarker, variations of BNP concentrations were associated with the re-hospitalization and mortality rates [26, 28]. Thus, a decrease in the BNP concentration on Day 4 relative to baseline was set as the UF-endpoint to determine its correlation with USCOM parameters. On Day 4, INO and SVRI on USCOM were found to be significantly correlated to a 30% reduction in BNP relative to baseline, and the predictive formula for the UF endpoint of a 30% reduction in BNP was: –2.462 + 0.028 × INO – 0.069 × SVRI (AUC ROC 0.831, 95% CI: 0.741–0.920; Hosmer-Lemeshow index 0.814), with a predicted sensitivity, specificity and accuracy of 70%, 83% and 75.0%, respectively, with a cut-off value of 0.567. INO, serving as one of the indicators of cardiac contractility, was also a distinctive hemodynamic parameter of USCOM [40]. Previous studies found that a higher INO could result in a greater SV under the same cardiac preload, while a lower INO was associated with impaired myocardial contractile function [41, 42]. SVRI reflected the cardiac afterload situation, with afterload being another key factor that influenced SV and CO. That is, with a reduction in SVRI, SV and CO were both increased. In the present trial, the elevation trends of INO, SV and CO, and a reduction trend of SVRI, were also observed during the UF process. Previous studies indicated that FTc effectively reflected the cardiac preload situation and was utilized to predict fluid responsiveness [43]. Chaiyakulsil *et al*. [19] employed USCOM, transthoracic echocardiography and electrical velocimetry for hemodynamic monitoring, and their findings confirmed that the FTc derived from the three non-invasive monitoring approaches could be utilized interchangeably. Moreover, FTc was easily measured by USCOM, and FTc may be a better predictive indicator to assess volume status and diuretic therapy [44]. As FTc was HR dependent, the present trial also confirmed a predictive formula of –2.640 – 0.088 × FTc – 0.036 × HR (AUC ROC 0.809, 95% CI: 0.709–0.909; Hosmer-Lemeshow index 0.655) for the UF endpoint of a 50% reduction in BNP, with a predicted cut-off value of 0.572, with sensitivity, specificity and accuracy for this model being 83%, 63% and 72.5%, respectively. Therefore, this trial highlights the feasibility of using USCOM indicators to predict the achievement of UF standards and has certain clinical significance.

However, the kinetics of BNP release vary between individuals and are influenced by factors such as renal function, medication use and comorbidities [45]. In some cases, despite improvements in hemodynamics, there may be a delayed reduction in BNP concentrations. This delay can be attributed to the time needed for the heart to adjust preload and afterload, as well as the clearance rate of BNP from the circulation [46]. In addition, it has also been reported that BNP cannot be used in isolation to measure congestion; rather, the concentration must be assessed in a proper clinical setting, like most other tests, and a precise cut-off point is not suitable. However, as noted by Mueller *et al*. [47], adjusting UF rates to patients’ vital signs and renal function has been linked to more effective decongestion and fewer heart failure events. It is well known that fluid overload is the main cause of hospitalization for patients with AHF, and changes in urine output may be explored as a predictor of fluid balance [48]. The present trial findings also indicated that urine output was positively correlated with the change rate of FTc (r = 0.255, *p* = 0.022) and negatively correlated with the VTI change rate (r = –0.27, *p* = 0.016). Thus, although in this trial, the rate of BNP reduction was used as a criterion for terminating UF, we need to consider other clinical features and the hemodynamic status of patients when considering BNP concentrations, including changes in fluid balance and signs of congestion, to make wise decisions regarding the termination of UF in clinical practice.

Another concern was the influence of comorbidities to hemodynamic changes during UF [49], since diabetes [50], hypertension [51], chronic kidney disease [52, 53] and cardiovascular diseases [54], both demonstrated to have an impact on fluid balance and vascular tone, are crucial factors in hemodynamics [55]. Patients with pre-existing cardiac conditions may be more susceptible to hemodynamic fluctuations during UF [56], and require careful monitoring and management. In contrast, another study demonstrated that the diverse physical conditions of critically ill patients might exert a rather limited influence on the graphic quality of USCOM [57]. In the present trial, the distribution was balanced between the UU group and the U group of patients with ADHF, so that it could be assumed that the hemodynamic impact was the same for both groups during their respective UF sessions.

This trial had several limitations. The selected ADHF patients were from a single center and were not stratified randomly, which might have led to a potential for bias. Besides the relative low number of samples, the frequent comorbidities might have had some influence on the hemodynamic changes in the graphic quality of USCOM and should be validated in a further large cohort trial. Since the kinetics of BNP release vary between individuals and are influenced by various factors, the usefulness of measurements of BNP concentrations may be limited by the possibility that their production and release may lag behind acute changes in hemodynamic measurements, thus additional potential endpoint criteria (e.g., urine output changes) should be explored in the near future. Further research is needed on the applicability of USCOM for different types of AHF patients (e.g., those with chronic kidney disease) and to validate its economic and clinical value after long-term follow-up data have been analyzed.

## 5. Conclusions

USCOM data were correlated significantly with UF outcomes and might serve as measures to determine endpoints for congestion therapy with UF in patients with ADHF. UF combined with USCOM also reduced the financial burdens of treatment and hospitalization for patients. No adverse events were reported due to USCOM within 24 h use, indicating that USCOM was a safe and non-invasive monitoring device.

## Data Availability

The datasets used and/or analyzed during the current study are available from the corresponding authors on reasonable request.

## Abbreviations

ADHF, acute decompensated heart failure; AHF, acute heart failure; AUC, area under curve; BNP, B-type natriuretic peptide; CI, confidence interval; CO, cardiac output; FTc, corrected flow time; HCT, hematocrit; HR, heart rate; INO, inotropy; IWRS, interactive web response system; MAP, mean arterial pressure; NT-proBNP, N-terminal pro-B-type natriuretic peptide; OR, odds ratio; ROC, receiver operating characteristic; SpO_2_, oxygen saturation; SV, stroke volume; SVI, stroke volume index; SVR, systemic vascular resistance; SVRI, systemic vascular resistance index; SVV, stroke volume variation; U, ultrafiltration alone; UF, ultrafiltration; USCOM, ultrasonic cardiac output monitor; UU, ultrafiltration + ultrasonic cardiac output monitor; VTI, velocity time integral.

## Supplementary Material

Supplementary material associated with this article can be found, in the online version, at https://doi.org/10.31083/RCM27100.
