# Supplementary material for: Cardiac Indices Parameters on the Ultrasonic Cardiac Output Monitor as Potential Indicators to Predict the Ultrafiltration Endpoint Success in Acute Heart Failure Treatment
Source: Rev Cardiovasc Med. 2025 May 22;26(5):27100. doi: 10.31083/RCM27100 (PMC12135639; doi:10.31083/RCM27100)
Supplement: Supplementary file 1 [file 2153-8174-26-5-27100-s1.docx]

**Supplementary Table 1. Hemodynamic indexes measured by UF in the U and UU groups and USCOM output in the UU group at 5 different time points.**

|  |  | **Day 1** | **Day 2** | **Day 3** | **Day 4** | **Day 7** | ***p*-value**  **(1-4 days)** | ***p-*value**  **(Day 7 vs. Day 4)** | ***p*-value** |
| --- | --- | --- | --- | --- | --- | --- | --- | --- | --- |
| **UF parameters of the U group** | SBP (mmHg) | 133.5 (117.3, 154.5) | 139.0 (115.0, 151.0) | 126.5 (109.0, 140.5) | 121.0 (107.5, 142.0) | 126.0 (108.5, 140.8) | 0.017 | 0.621 | 0.010 |
|  | DBP (mmHg) | 70.0 (64.0, 81.3) | 71.0 (66.5, 78.5) | 67.0 (61.8, 70.8) | 65.0 (58.8, 74.0) | 64.5 (58.5, 73.3) | 0.001 | 0.726 | <0.001 |
|  | MAP (mmHg) | 94.8 (83.3, 104.5) | 95.8 (85.3, 100.5) | 88.7 (78.8, 94.0) | 85.8 (76.5, 99.2) | 85.5 (75.5, 94.3) | <0.001 | 0.634 | <0.001 |
|  | HR (bpm) | 82.0 (72.0, 91.8) | 80.5 (72.3, 90.3) | 77.0 (64.3, 85.3) | 75.0 (63.3, 86.3) | 74.5 (64.0, 83.8) | 0.058 | 0.649 | 0.070 |
|  | Urine output (mL) | 950.0 (700.0, 1,362.5) | 1,750.0 (1,550.0, 2,525.0) | 2,250.0 (1,575.0, 2,850.0) | 1,900.0 (1,356.25, 2,675.0) | 1,500.0 (1,300.0, 1,700.0) | <0.001 | <0.001 | 0.045 |
|  | SpO_2_ (%) | 96.0 (95.0, 97.0) | 96.5 (96.0, 98.0) | 97.0 (96.0, 98.0) | 97.0 (96.75, 98.0) | 97.5 (97.0, 98.0) | 0.004 | 0.359 | <0.001 |
|  | HCT (%) | 35.0 (33.1, 36.3) | 35.1 (32.8, 36.6) | 35.8 (32.9, 37.8) | 36.2 (33.2, 38.2) | 36.3 (34.3, 37.9) | 0.004 | 0.431 | <0.001 |
|  | BNP (pg/mL) | 2,098.9 (709.7, 3,015.8) | 1,434.5 (654.4, 2,449.6) | 1,127.5 (669.9, 1,972.5) | 758.3 (503.5, 1,303.6) | 534.0 (418.7, 972.2) | <0.001 | 0.007 | <0.001 |
| **UF**  **parameters of the UU group** | SBP (mmHg) | 128.5 (111.2, 148.0) | 126.5 (113.0, 137.0) | 124.5 (110.5, 133.0) | 122.0 (105.2, 138.0) | 117.5 (105.8, 134.5) | 0.002 | 0.492 | 0.001 |
|  | DBP (mmHg) | 66.5 (63.8, 81.0) | 68.5 (62.5, 80.0) | 63.5 (61.8, 71.2) | 61.5 (60.0, 69.2) | 62.5 (61.0, 71.8) | <0.001 | 0.911 | <0.001 |
|  | MAP (mmHg) | 90.2 (81.0, 98.7) | 86.2 (80.8, 98.2) | 86.7 (77.9, 91.6) | 83.5 (74.8, 89.8) | 84.5 (75.4, 91.3) | <0.001 | 0.720 | <0.001 |
|  | HR (bpm) | 84.5 (71.8, 106.2) | 84.5 (74.8, 95.2) | 76.3 (70.5, 92.8) | 77.5 (70.0, 93.2) | 81.0 (69.8, 91.0) | 0.184 | 0.755 | 0.140 |
|  | Urine output (mL) | 895.0 (700.0, 1,000.0) | 1,850.0 (1,600.0, 2,462.5) | 2,200.0 (1,787.5, 2,875.0) | 2,050.0 (1,737.5, 2,425.0) | 1,725.0 (1,300.0, 1,862.5) | <0.001 | <0.001 | 0.042 |
|  | SpO_2_ (%) | 96.0 (95.0, 97.0) | 97.0 (96.0, 98.0) | 97.0 (96.8, 98.0) | 97.0 (96.0, 98.2) | 97.5 (97.0, 98.0) | 0.001 | 0.616 | <0.001 |
|  | HCT (%) | 35.9 (33.6, 38.1) | 35.5 (34.0, 39.0) | 37.0 (35.3, 38.3) | 38.0 (36.1, 39.3) | 38.0 (36.8, 39.2) | <0.001 | 0.466 | <0.001 |
|  | BNP (pg/mL) | 1,647.0 (1,047.0, 4,041.3) | 1,063.5 (686.2, 2,869.9) | 980.7 (722.2, 2,523.0) | 738.0 (609.0, 1,801.6) | 615.0 (402.2, 1,308.0) | <0.001 | 0.007 | <0.001 |
| **USCOM monitoring output of the UU group** | SV (mL) | 22.3 (14.1, 33.5) | 27.6 (18.6, 34.6) | 32.6 (27.4, 40.3) | 35.1 (23.8, 50.1) | 40.3 (26.7, 49.6) | <0.001 | 0.279 | <0.001 |
|  | SVI (mL/beats) | 12.5 (7.8, 18.8) | 14.2 (10.8, 18.8) | 17.8 (13.2, 21.4) | 19.1 (12.3, 30.1) | 22.7 (13.4, 27.5) | <0.001 | 0.250 | <0.001 |
|  | SVV (%) | 58.5 (34.5, 72.5) | 56.5 (40.5, 67.2) | 51.2 (31.5, 78.0) | 52.5 (33.2, 73.5) | 52.0 (35.2, 62.8) | 0.147 | 0.505 | 0.367 |
|  | CO (L/min) | 1.9 (1.6, 2.3) | 2.1 (1.6, 2.8) | 2.9 (1.9, 3.3) | 2.8 (2.2, 3.4) | 3.2 (2.3, 3.6) | <0.001 | 0.027 | <0.001 |
|  | Cardiac index (L/min/m^2^) | 1.1 (0.8, 1.3) | 1.1 (0.9, 1.5) | 1.4 (1.1, 1.8) | 1.5 (1.2, 1.8) | 1.7 (1.3, 2.0) | <0.001 | 0.020 | <0.001 |
|  | INO (W/m^2^) | 0.6 (0.4, 0.9) | 0.7 (0.6, 1.0) | 0.8 (0.6, 1.0) | 0.9 (0.7, 1.2) | 0.9 (0.8, 1.3) | <0.001 | 0.009 | <0.001 |
|  | SVRI (mmHg·min/mL) | 8,339 (6,356, 13,527) | 6,759 (5,664, 11,614) | 5,799 (4,369, 9,988) | 4,788 (3,998, 8,234) | 4,304 (3,799, 7,258) | <0.001 | <0.001 | <0.001 |
|  | SVR (mmHg·min/L) | 4,430 (3,258, 7,862) | 3,585 (2,933, 5,790) | 3,098 (2,368, 5,077) | 2,616 (2,216, 4,578) | 2,420 (1,918, 3,811) | <0.001 | <0.001 | <0.001 |
|  | FTc (ms) | 427 (388, 464) | 403 (348, 432) | 367 (331, 413) | 328 (310, 405) | 313 (279, 380) | <0.001 | <0.001 | <0.001 |
|  | VTI (cm) | 8.1 (6.4, 9.4) | 9.3 (7.5, 11.1) | 10.6 (9.3, 13.0) | 12.1 (9.7, 14.8) | 13.8 (11.0, 16.6) | <0.001 | <0.001 | <0.001 |

Data are presented as median (Q1, Q3) unless otherwise indicated.

Abbreviations: BNP, B-type natriuretic peptide; CO, cardiac output; DBP, diastolic blood pressure; FTc, corrected flow time; HCT, hematocrit; HR, heart rate; INO, inotropy; MAP, mean arterial pressure; SBP, systolic blood pressure; SpO_2_, oxygen saturation; SV, stroke volume; SVI, stroke volume index; SVR, systemic vascular resistance; SVRI, systemic vascular resistance index; SVV, stroke volume variation; UF, ultrafiltration; USCOM, ultrasonic cardiac output monitor; U group, ultrafiltration group; UU group, ultrafiltration + ultrasonic cardiac output monitor group; VTI, velocity time integral.

**Supplementary Table 2.** **Correlations between the** **changes of USCOM output and UF parameter on Day 4 compared to baseline of UU treatments (correlation coefficient/*p*-value).**

|  |  | **MAP** | **HR** | **Urine output** | **SpO_2_** | **HCT** | **BNP** |
| --- | --- | --- | --- | --- | --- | --- | --- |
| **SV** | r | -0.329 | 0.673 | -0.186 | 0.229 | 0.106 | -0.165 |
|  | *p* | **0.003** | **<0.001** | 0.099 | **0.041** | 0.348 | 0.143 |
| **SVI** | r | -0.329 | 0.673 | -0.186 | 0.229 | 0.106 | -0.165 |
|  | *p* | **0.003** | **<0.001** | 0.099 | **0.041** | 0.348 | 0.143 |
| **SVV** | r | 0.296 | 0.043 | 0.052 | -0.196 | -0.047 | 0.040 |
|  | *p* | **0.008** | 0.702 | 0.607 | 0.081 | 0.679 | 0.724 |
| **CO** | r | -0.203 | -0.163 | -0.192 | 0.145 | 0.071 | -0.046 |
|  | *p* | 0.072 | 0.148 | 0.088 | 0.201 | 0.532 | 0.686 |
| **Cardiac index** | r | -0.202 | -0.163 | -0.192 | 0.145 | 0.071 | -0.046 |
|  | *p* | 0.072 | 0.148 | 0.088 | 0.199 | 0.532 | 0.684 |
| **INO** | r | -0.204 | -0.125 | 0.005 | 0.042 | 0.232 | -0.473 |
|  | *p* | 0.070 | 0.269 | 0.964 | 0.714 | 0.038 | **<0.001** |
| **SVRI** | r | 0.322 | 0.012 | 0.189 | -0.043 | 0.251 | 0.422 |
|  | *p* | **0.004** | 0.917 | 0.094 | 0.668 | **0.012** | **<0.001** |
| **SVR** | r | 0.322 | 0.012 | 0.189 | -0.043 | 0.251 | 0.422 |
|  | *p* | **0.004** | 0.917 | 0.094 | 0.668 | **0.012** | **<0.001** |
| **FTc** | r | 0.280 | 0.203 | 0.255 | -0.198 | -0.500 | 0.353 |
|  | *p* | **0.012** | 0.071 | **0.022** | **0.048** | **<0.001** | **0.001** |
| **VTI** | r | -0.263 | -0.454 | -0.27 | 0.197 | -0.137 | -0.602 |
|  | *p* | **0.019** | **<0.001** | **0.016** | **0.050** | 0.174 | **<0.001** |
| **HR** | r | 0.273 | / | -0.123 | -0.222 | -0.306 | 0.229 |
|  | *p* | **0.014** | / | 0.277 | **0.048** | 0.753 | **0.041** |

Note: The bolded data represent a p-value less than 0.05.

Abbreviations: BNP, B-type natriuretic peptide; CO, cardiac output; FTc, corrected flow time; HCT, hematocrit; HR, heart rate; INO, inotropy; MAP, mean arterial pressure; SpO_2_, oxygen saturation; SV, stroke volume; SVI, stroke volume index; SVR, systemic vascular resistance; SVRI, systemic vascular resistance index; SVV, stroke volume variation; UF, ultrafiltration; USCOM, ultrasonic cardiac output monitor; UU, ultrafiltration + ultrasonic cardiac output monitor; VTI, velocity time integral.

**Supplementary Table 3. Statistics based on a 30% reduction on Day 4 in BNP from the baseline during UF and USCOM output.**

| **Predictive index (change rates)** | **Formula (probability value, *p*)** | **Cut-off value** | **AUC (95% CI)** | **Sensitivity (%)** | **Specificity (%)** | **Accuracy (%)** | **Hosmer-Lemeshow index** |
| --- | --- | --- | --- | --- | --- | --- | --- |
| INO | -0.712 + 0.038 × INO | 0.480 | 0.773 (0.665-0.881) | 68 (34, 90) | 77 (47, 90) | 73.8 | 0.142 |
| SVRI | -1.922 - 0.081 × SVRI | 0.467 | 0.779 (0.678-0.879) | 64 (37, 87) | 73 (43, 97) | 72.5 | 0.520 |
| FTc | -0.648 - 0.08 × FTc | 0.407 | 0.708 (0.592-0.823) | 64 (34, 84) | 73 (37, 90) | 65.0 | 0.564 |
| VTI | -0.506 + 0.028 × VTI | 0.340 | 0.703 (0.583-0.822) | 56 (33, 86) | 60 (42, 90) | 70.0 | 0.018 |
| SVRI + INO | -2.462 + 0.028 × INO - 0.069 × SVRI | 0.567 | 0.831 (0.741-0.920) | 70 (37, 89) | 83 (47, 97) | 75.0 | 0.814 |
| INO + FTc | -1.51 + 0.032 × INO - 0.064 × FTc | 0.527 | 0.799 (0.700-0.899) | 68 (36, 97) | 80 (49, 95) | 72.5 | 0.654 |
| INO + VTI | -1.673 + 0.027 × VTI + 0.038 × INO | 0.480 | 0.804 (0.708-0.900) | 66 (39, 89) | 34 (11, 61) | 76.3 | 0.287 |

Abbreviations: AUC, area under curve; BNP, B-type natriuretic peptide; CI, confidence interval; FTc, corrected flow time; INO, inotropy; SVRI, systemic vascular resistance index; UF, ultrafiltration; USCOM, ultrasonic cardiac output monitor; VTI, velocity time integral.

**Supplementary Table 4. Statistics based on a 50% reduction on day 4 in BNP from the baseline during UF and USCOM output.**

| **Predictive index**  **(change rates)** | **Formula (probability value, *p*)** | **Cut-off value** | **AUC (95% CI)** | **Sensitivity (%)** | **Specificity (%)** | **Accuracy (%)** | **Hosmer-Lemeshow test** |
| --- | --- | --- | --- | --- | --- | --- | --- |
| INO | -1.603 + 0.016 × INO | 0.439 | 0.699 (0.582-0.815) | 72 (35, 96) | 58 (31, 80) | 72.5 | 0.090 |
| SVRI | -2.571 - 0.049 × SVRI | 0.398 | 0.709 (0.577-0.840) | 74 (46, 87) | 60 (29, 84) | 68.8 | 0.247 |
| FTc | -2.482 - 0.09 × FTc | 0.475 | 0.749 (0.632-0.866) | 83 (44, 96) | 63 (32, 83) | 68.8 | 0.260 |
| VTI | -2.002 + 0.025 × VTI | 0.364 | 0.719 (0.599-0.838) | 70 (46, 91) | 58 (31, 84) | 72.5 | 0.394 |
| HR | -1.096 - 0.037 × HR | 0.388 | 0.722 (0.594-0.850) | 74 (48, 87) | 60 (29, 85) | 73.8 | 0.999 |
| Urine output | 0.581 - 0.012 × urine output | 0.373 | 0.700 (0.562-0.838) | 65 (45, 82) | 64 (28, 90) | 73.8 | 0.677 |
| INO + FTc | -3.113 -0.087 × FTc + 0.015 × INO | 0.476 | 0.780 (0.665-0.894) | 78 (48, 96) | 61 (33, 84) | 78.8 | 0.903 |
| INO + HR | -1.702 - 0.035 × HR + 0.014 × INO | 0.458 | 0.754 (0.629-0.880) | 83 (52, 87) | 63 (30, 86) | 77.5 | 0.281 |
| INO + Urine output | -0.077 + 0.016 × INO -0.012 × urine output | 0.485 | 0.741 (0.611-0.870) | 78 (52, 87) | 61 (30, 86) | 76.3 | 0.586 |
| SVRI + HR | -3.034 - 0.056 × SVRI -0.042 × HR | 0.484 | 0.786 (0.680-0.893) | 78 (48, 96) | 61 (33, 84) | 75.0 | 0.681 |
| SVRI + Urine output | -1.103 - 0.011 × urine output - 0.046 × SVRI | 0.477 | 0.764 (0.635-0.892) | 78 (57, 87) | 61 (30, 88) | 77.5 | 0.141 |
| FTc + VTI | -2.999 -0.073 × FTc + 0.018 × VTI | 0.493 | 0.774 (0.658-0.891) | 83 (48, 91) | 63 (32, 84) | 75.0 | 0.600 |
| FTc + HR | -2.640 - 0.088 × FTc - 0.036 × HR | 0.572 | 0.809 (0.709-0.909) | 83 (52, 96) | 63 (33, 86) | 72.5 | 0.655 |
| VTI + Urine output | 0.022 × VTI - 0.01 × urine output | 0.493 | 0.759 (0.640-0.878) | 74 (52, 91) | 60 (32, 86) | 73.8 | 0.391 |
| Urine output + HR | 0.411 - 0.012 × urine output - 0.036 × HR | 0.502 | 0.785 (0.666-0.903) | 78 (52, 91) | 61 (32, 86) | 75.0 | 0.029 |

Abbreviations: AUC, area under curve; BNP, B-type natriuretic peptide; CI, confidence interval; FTc, corrected flow time; HR, heart rate; INO, inotropy; SVRI, systemic vascular resistance index; UF, ultrafiltration; USCOM, ultrasonic cardiac output monitor; VTI, velocity time integral.

**
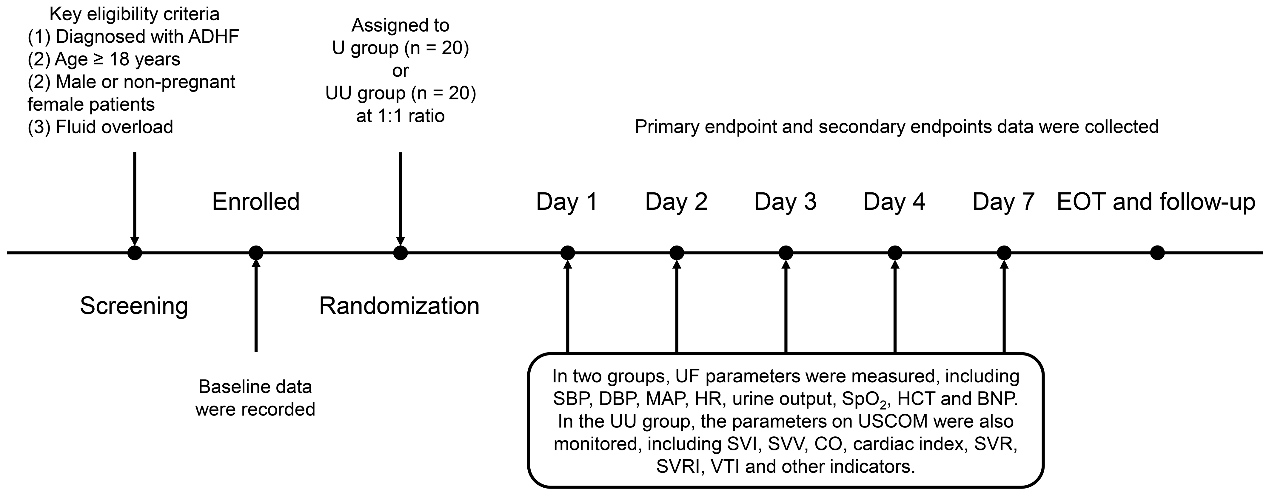
**

**Supplementary Fig. 1. Study design.**

ADHF, acute decompensated heart failure; BNP, B-type natriuretic peptide; CO, cardiac output; DBP, diastolic blood pressure; EOT, end of treatment; HCT, hematocrit; HR, heart rate; MAP, mean arterial pressure; SBP, systolic blood pressure; SpO_2_, oxygen saturation; SVI, stroke volume index; SVR, systemic vascular resistance; SVRI, systemic vascular resistance index; SVV, stroke volume variation; UF, ultrafiltration; USCOM, ultrasonic cardiac output monitor; U group, ultrafiltration group; UU group, ultrafiltration + ultrasonic cardiac output monitor group; VTI, velocity time integral.
